# Influences on Dietary Choices during Day versus Night Shift in Shift Workers: A Mixed Methods Study

**DOI:** 10.3390/nu9030193

**Published:** 2017-02-26

**Authors:** Emily K. Bonnell, Catherine E. Huggins, Chris T. Huggins, Tracy A. McCaffrey, Claire Palermo, Maxine P. Bonham

**Affiliations:** 1 Department of Nutrition, Dietetics and Food, Level 1, 264 Ferntree Gully Road, Monash University, Melbourne, Notting Hill VIC 3168, Australia; emilykbonnell@gmail.com (E.K.B.); Catherine.huggins@monash.edu (C.E.H.); tracy.mccaffrey@monash.edu (T.A.M.); claire.palermo@monash.edu (C.P.); 2 Department of Community Emergency Health and Paramedic Practice, Monash University, Frankston VIC 3199, Australia; chris.huggins@monash.edu

**Keywords:** nutrition, shift work, communicative disease, qualitative methodology

## Abstract

Shift work is associated with diet-related chronic conditions such as obesity and cardiovascular disease. This study aimed to explore factors influencing food choice and dietary intake in shift workers. A fixed mixed method study design was undertaken on a convenience sample of firefighters who continually work a rotating roster. Six focus groups (*n* = 41) were conducted to establish factors affecting dietary intake whilst at work. Dietary intake was assessed using repeated 24 h dietary recalls (*n* = 19). Interviews were audio recorded, transcribed verbatim, and interpreted using thematic analysis. Dietary data were entered into FoodWorks and analysed using Wilcoxon signed-rank test; *p* < 0.05 was considered significant. Thematic analysis highlighted four key themes influencing dietary intake: shift schedule; attitudes and decisions of co-workers; time and accessibility; and knowledge of the relationship between food and health. Participants reported consuming more discretionary foods and limited availability of healthy food choices on night shift. Energy intakes (kJ/day) did not differ between days that included a day or night shift but greater energy density (ED_energy_, kJ/g/day) of the diet was observed on night shift compared with day shift. This study has identified a number of dietary-specific shift-related factors that may contribute to an increase in unhealthy behaviours in a shift-working population. Given the increased risk of developing chronic diseases, organisational change to support workers in this environment is warranted.

## 1. Introduction

Compared to day workers, shift workers are at a higher risk of many diet-related chronic conditions, including obesity [1,2], cardiovascular disease (CVD) [3,4], and type 2 diabetes [5,6]. Across the world, the proportion of the workforce who engage in shift work varies, ranging from around 10% in Brazil to 16% in Australia, 24% in Czech Republic, and greater than 50% in Jamaica [7].

Shift work that includes overnight shifts disrupts the circadian biological clock governing the body’s internal regulation of sleep and wake times, which in turn affects energy metabolism and may promote weight gain [8]. During nighttime sleep, the body is in a fasting state promoting release of stored glucose and relative insulin resistance (compared with day) to permit preferential usage of glucose by the central nervous system rather than for muscle energy [9,10]. Eating during nocturnal hours, when the body is programmed to be asleep, disrupts the metabolic milieu [11]. Acute experimental studies have found that a meal eaten at night generates an exaggerated glucose and lipid response compared with the same meal eaten during the day [12]. Long-term excursions in glucose and lipids are risk factors for cardiovascular disease [13].

Eating at work during night shift is, therefore, a modern-day risk factor for cardiovascular disease.

Although shift workers have a propensity for snacking at night [14] and making poor food choices [15], these habits do not appear to be at the expense of an increased energy intake. A recent systematic review and meta-analyses reporting on a total of 12 studies with 10,367 day workers and 4726 shift workers concluded no difference in energy intake between shift workers and their daytime counterparts [16]. These findings suggest that meal timing and food choice at night, rather than energy intake per se, is a key contributor to the increased risk of CVD observed in shift workers and imply that shift workers eat a substantial proportion of their meals during a time of suboptimal glucose and lipid tolerance. Recommending complete avoidance of food at night is unrealistic for many shift workers. Thus, the development of strategies to minimise the metabolic disturbance to food intake at night are required. These strategies will rely, in part, on an understanding of dietary practices of shift workers. Physiological, psychosocial, environmental, and organisational influences were identified as the main themes affecting food choices in a qualitative study of Australian paramedics (an essential shift work profession), however, shift-specific factors (i.e., night shift vs. day shift) were not explored [17]. Understanding shift-specific factors is important to permit development of dietary advice that is practical and feasible for shift workers.

Using a fixed mixed methods approach, the aim of this study was to: (1) explore, in rotating shift workers, factors influencing dietary intake on day shift and on night shift; (2) assess the types of foods and drinks consumed and the timing of each eating occasion on work days. A mixed method approach was taken to facilitate a more complete understanding of food choices and the factors affecting these choices.

## 2. Materials and Methods

### 2.1. Design

A fixed mixed method study design was undertaken (i.e., the decision to undertake both qualitative and quantitative methods was made before the research is started), with concurrent data collection methods [18]. Focus groups were conducted to establish the factors affecting dietary intake of rotational shift workers whilst at work and quantitative assessment of their dietary intake using 24 h dietary recalls. Ethics approval for this study was granted by Monash University Human Research Ethics Committee (CF14/1491-2014000703) and signed informed consent was obtained from all participants.

### 2.2. Sampling

A convenience-based sample of firefighters from Melbourne provided a population of rotating shift workers for the study. Potential participants were a mixture of recruit firefighters (<1 year) or experienced firefighters. All participants were provided with information about the aims of the research study by the researcher face to face at the beginning of a 2-day mandatory training course and then were invited to participate in the focus group and/or dietary assessment after the completion of their training. This sample followed a 10/14 rotating shift schedule: two consecutive 10 h day shifts followed by two consecutive 14 h night shifts, then four rostered days off. Recruitment continued until the female researcher (EB) determined that further discussions would not disclose new information [19].

### 2.3. Data Collection

Participants were required to self-report their age, height, and weight (current weight and weight on starting shift work). Body mass index (BMI) was calculated from body height and weight in kg/m^2^.

Focus groups were held at the training facility of the employer and facilitated by the same student researcher (EB) using a list of semi-structured open questions (Table 1) developed by the research team. EB is also an accredited practising dietitian with research training in qualitative methods. The approach was pragmatic, aiming to elicit information that would support understanding of data collected from quantitative methods. Participants were prompted to discuss factors that influenced their food choices at work and each focus group lasted between 40 min and 1 h. Each group was audio-recorded and transcribed verbatim by the student researcher (EB) with confidentiality maintained. Transcripts were completed within the week following each focus group to allow a determination to be made whether data saturation had been reached and thus whether recruitment of new focus groups should be halted. Transcripts were not returned to participants for comment.

Dietary intakes were assessed using an adapted version of the United States Department of Agriculture (USDA) multiple-pass 24 h recall method [20]. Two recalls covered 24 h periods during which an entire night shift was worked (for example, midday Thursday to midday Friday, encompassing the 1700–0700 h night shift) and two covered equivalent 24 h periods encompassing the entire day shift (0700–1700 h). As food intake can vary from day to day, the paired repetition of 24 h recalls allowed the capture of food intake on different days of the week. All dietary recalls were conducted over the telephone by the same researcher (EB). Participants were provided with an adapted copy of the 4000 for Health food model booklet on enrolment into the study [21], which provided visual examples of meal portions and mug and cup sizes, to assist them to describe the quantity of food and beverages consumed.

### 2.4. Data Analysis

Data from the dietary intake and focus groups were analysed concurrently to look for congruence and difference. Quantitative data were used to assist in the interpretation of qualitative data and to verify its accuracy.

A manual thematic analysis was undertaken using qualitative description [22] with simple depiction of the factors influencing shift workers’ food intake. Transcript data were initially analysed by the first author (EB). The texts were coded and then grouping codes were used to identify common ideas and themes. Two focus groups were independently analysed (by CP) using the same approach. The themes identified independently were then discussed by both authors and agreement sought on final themes. Descriptors with illustrative quotes were selected to aid interpretation. Representative quotes are presented from each focus group rather than individually.

Only participants who completed two or more dietary recalls (at least one day shift and one night shift) were included in the dietary analysis. Dietary data were entered into FoodWorks (Xyris Software, Brisbane, Australia). Energy, macronutrient, and fibre intakes were calculated based on food composition data available from Nutrient Tables (NUTTAB) 2011 (Food Standards Australia and New Zealand) and Australian Food, Supplement and Nutrient Database (AUSNUT) 2013 (Food Standards Australia and New Zealand). Energy density (ED, kJ/g/day) was calculated in three ways [23]: ED of all food and beverages (EDall); ED of solid foods only (EDsolid), and ED of all solid foods plus soups, milk as food, milk as a drink, and beverages containing >21 kJ/100 g (EDenergy). Eating occasions during each 24 h recall were described using participants’ definitions (e.g., breakfast, lunch, dinner, snack and beverage, or beverage only). Lunch and dinner meals were further classified into three categories: cooked meals, takeaway meals, and easy-to-prepare meals (sandwiches, rolls, wraps, salads, or heat-and-serve dishes (e.g., microwave rice) based on examples provided in the focus groups. Snacks were classified into food groups based on the Australian dietary guidelines [24].

Descriptive statistics were calculated using Statistical Packages for the Social Sciences (SPSS; IBM SPSS Statistics for Windows, Version 23.0. Armonk, NY, USA; IBM Corp.). Data were tested for normality using the Shapiro–Wilk test, and reported as median (25th and 75th percentiles). Wilcoxon signed-rank test was used to compare dietary intake between 24 h periods where a day shift was worked compared to a night shift. Differences in dietary intake during hours at work were also compared (i.e., day shift (10 h) versus night shift (14 h)) using the Wilcoxon signed-rank test. Significance was reported as *p* < 0.05.

## 3. Results

### 3.1. Participation

Fifty-five potential participants attended the mandatory training program during the data collection period. Forty-one took part in six focus groups (*n* = 8, *n* = 7, *n* = 7, *n* = 4, *n* = 12, and *n* = 3 participants, respectively) and participant characteristics are presented in Table 2. Recruit firefighters and experienced firefighters were in different focus groups due to the scheduling of the training. The median (25th, 75th percentile) years working rotating shift work was 6 (0.58, 29). A total of 29 people consented to participate in the dietary intake collection, all but one of whom participated in the focus groups. Fifteen participants completed four dietary recalls and four competed two or more, and thus 19 were included in the dietary analysis. The other 10 participants were excluded as follows: *n* = 9 could not be contacted during the study period (on annual leave, incorrect contact details, unresponsive to contact attempts) and *n* = 1 only completed one dietary recall. Participants represented 27 of the 47 fire stations across metropolitan Melbourne.

### 3.2. Factors Affecting Dietary Intake

Four key themes related to factors influencing dietary intake emerged from the focus groups (Table 3) and include shift schedule; attitudes and decisions of co-workers; time and accessibility; and knowledge of the relationship between food and health. Each theme was connected to the workplace and included social (attitudes and choices of others), cultural (demands of daily tasks), and physical factors (environmental) that affect decision making around food choices. Dietary data that support the emergent themes are presented at the end of each relevant Theme, with differences between night shift and days shift emphasised where evident.

#### 3.2.1. Theme 1: Shift Schedule Influences Types of Meals and Snacks Consumed at Work

Participants reported that irrespective of shift, the main meal consumed at work might include: communal meals cooked at the workplace, takeaway food purchased during shifts, or food brought from home. Lunch intake differed from dinner. Four of the six focus groups reported that for lunch they would choose an easy-to-prepare meal, such as meat and salad rolls or wraps, roast rolls, and schnitzel rolls. Their communal evening meals generally took much more time to prepareand they placed much higher value on these dinner meals:

“You pride yourself on what you cook for dinner so yeah. Lot more effort goes into dinner.”

—Focus group 5

For takeaway foods, participants stated that they would more often choose foods considered to be healthy when they were on day shift than when they were on night shift. They also consumed takeaway foods more often on night shift. This was partly due to the presence of established routines at particular fire stations where takeaway meals were consumed every second night shift (Focus groups 2–4), every Friday night (Focus groups 1, 2, 4, 6), or over a weekend shift (Focus group 6).

“Night shift: fish and chips or takeaway. Day shift I generally try and keep that clean and go to the supermarket.”

—Focus group 3

Lack of time for preparing food to take to work was highlighted as an important reason for purchasing takeaway food during night shift. Some firefighters worked other jobs on their days off and/or during the day before beginning their night shift. Others had family commitments (young families) that prevented them preparing their own food:

“At work sometimes it is hard for me to bring stuff in if I’m on the nightshift sometimes because of what I’ve been doing, looking after the kids during the day or whatever. So it’s convenient to just get takeaway when you’re at work.”

—Focus group 4

Participants noted that other meals provided to them by their brigade when they were on call were usually unhealthy regardless of shift schedule:

“I think one disadvantage with this organisation is when we go to fire duties the organisation sends out high fat foods. They send out pizza, pies, soft drinks and stuff like that.”

—Focus group 3

The majority of work snacks were brought from home. Irrespective of shift schedule, these were mainly fruit, nuts, and yoghurt. Some participants said that they were more likely to eat chocolates and ice cream, supplied at the workplace, after dinner (on a night shift) than when they were on a day shift; other groups indicated no difference. However, workers indicated that they would eat chocolates and sweet biscuits after an extended night call because nothing else was available.

“You’d start eating a packet of biscuits coz that’s the only thing around at the station to eat, yeah so that’s the worst part…night shifts where you do go on call, you come back and…you don’t plan on eating as such but you (have) got to basically or you starve.”

—Focus group 2

**Figure 1:**
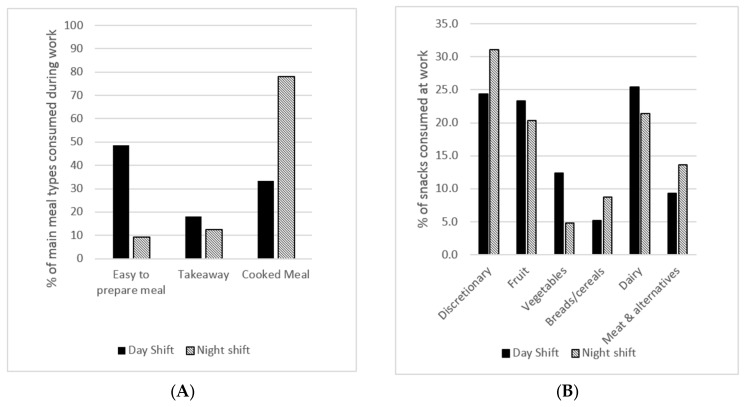
Comparison of main meal and food groups consumed by rotating shift workers (*n* = 19) comparing dietary intake from day shift and night shift: (**A**) Categories of main meals were identified during focus groups. Easy-to-prepare meals included sandwiches, rolls, wraps, or salads, or heat-and-serve dishes (e.g., microwave rice); (**B**) snacks were categorised according to the Australian Guide to Healthy Eating food groups (*n* = 296 snack foods consumed over 70 snacking occasions).

A deductive examination of the dietary data for the frequency of main meal types (Figure 1A) and snacks (Figure 1B) consumed at work supports the findings of the focus groups. On night shift, compared with day shift, a greater proportion of discretionary snack foods were consumed comprising mostly of chocolates, ice creams, and sweet pastries (Figure 1B).

Energy intakes (kJ/day) were not different between day or night shift (Table 4). A significantly higher percentage energy from sugar was observed during the 24 h period that included a night shift compared with the 24 h period that included a day shift (*p* = 0.036). There were fewer eating occasions, and a greater overall energy density (ED_energy_, kJ/g/day) of the diet during a 24 h period that included a night shift compared with a day shift period (*p* < 0.05, Table 4).

#### 3.2.2. Theme 2: Dietary Intake Is Affected by the Dietary Choices and Attitudes of Co-Workers

Social influence was reported as a major factor influencing dietary choices at work and in some cases, carried over to impact choices made at home.

“Most things that we do we generally do with someone else…it’s a pretty social job so you’re generally…sitting around (with) other people so you do a lot of stuff together.”

—Focus group 5

Selection of meals purchased or cooked communally at lunch and dinner tended to be a majority decision, with group consensus valued over individual preference. This could have both a positive and negative impact on dietary intake.

“You don’t want to be the odd one out, so if everyone wants to get salad rolls you’re hardly gonna (sic) say well no I want Maccas (McDonalds) or I want pizza, but then if like 2 or 3 guys go ‘ohh let’s get pizza tonight boys’ you don’t want to be the guy who goes nah I’d rather have a salad roll so… it’s a group decision.”

—Focus group 5

“Couple of guys you know you’ll say ‘alright we’ll have a salad tonight’ and they’ll just laugh at you kind of thing. So you gotta (sic), if you’re gonna (sic) cook for the station you gotta (sic) cook for everyone.”

—Focus group 2

The choice to purchase takeaway food on shift was influenced by others. Participants explained that the crew must accompany one another to purchase food in case they are called out during this time. The temptation then arises to purchase what others are getting, neglecting an often-healthier choice brought from home.

“If we go out and someone else has to buy lunch and I like what they’re getting, I’ll forget what I took into work and I’ll say ‘I’ll have some of that!’ That’s how I have more takeaway.”

—Focus group 1

The strongest predictor of whether meals were cooked communally was the presence of somebody interested in food and cooking, which tended to encourage others to cook as well. If this person then left the station, motivation within the crew faded:

“We had a guy come from ah from out of town…and he enjoyed cooking, so for that whole month that he was with us he was cooking meals, motivated the guys and that lasted probably two or three months (afterwards) and then ended up going to get takeaway food that was ah around the back of the station.”

—Focus group 5

These participants also initially felt peer pressure to become involved in communal cook-ups:

“If you’re at a station where they do cook together there’s a pretty big culture around it so you don’t want to be a splitter and not eat with them so yeah, you tend to have bigger meals and maybe not as healthy because you don’t want to not eat with them.”

—Focus group 2

#### 3.2.3. Theme 3: Food Choices Are Dependent on the Availability of Time and Ease of Accessibility

Accessibility to food was reported to influence food choices. Convenience and temptation were highlighted as major reasons for non-hungry eating at work. During two focus groups, it was reported that eating was a way to combat boredom on quiet work days. The majority of non-hungry eating occasions involved discretionary items freely available at the workplace.

“It’s always a battle because they’re there (chocolates). We never have that stuff at home so…the opportunity is there, which is quite tempting.”

—Focus group 6

The type of takeaway bought on shift was reported to be largely dependent on the location of the station and whether convenience food stores were close by. Participants described challenges obtaining food when they were “moved up”—that is, when a crew shifts to cover another station that is out on an extended call. Workers described snacking on chocolates and ice creams available at the station because they either did not have an opportunity to prepare a meal or they had left it behind at their home station. The drive back to their home station or during shifts with repeated call-outs were identified as situations where they might stop to purchase something quick, easy, and often of poor nutritional quality, depending on the length and timing of the move-up or call-outs.

“When you get a move up because you don’t know how long (it) is going to be…it’s getting past 3 o’clock (a.m.)…you’re getting hungry and you’re like, ‘Oh crap, I will have that Magnum (ice-cream bar)’ …and you do go and buy something crap because it’s been that long since you’ve had anything to eat.”

—Focus group 3

“We had a heap of calls in a row, we had a fire, a couple of false alarms and something else and by the time we got back to the station it was about 9.30 (p.m.) and I think (colleague) and myself both had about three goes at cooking our dinner and mine ended up in the bin…we walked up to McDonalds on the corner.”

—Focus group 4

Participants varied in the foods they chose when tired after a busy shift. One group noted that the day after a busy night shift they were more likely to choose takeaway foods or easy-to-prepare meals, because they were too tired to put effort into cooking:

“After I’ve finished a night shift I’ll often, like first two days after, I’ll just go and get Subway for lunch or something coz I can’t’ be bothered putting the same effort into preparing my food as when I feel fully ah, revitalised.”

—Focus group 5

Meals might be delayed by calls or in some instances missed all together. For example, breakfast following night shift might be missed entirely if the end of shift was delayed, as this was not designated as a mealtime. As each shift is unpredictable, the participants explained that they prepare for calls and busy shifts by eating when the opportunity arises, with the expectation that this could be their only chance to eat during the rest of the shift.

“You (might) get delayed in the morning and you don’t get breakfast. You get a call. The next thing you know by the time you get home it’s half past 10 or 11 o’clock in the morning and the last time you ate was at 6.30 the night before.”

—Focus group 3

“Even though you’re not hungry, you will eat because you know you could have a bad night.”

—Focus group 3

#### 3.2.4. Theme 4: Firefighters Endeavour to Make Healthy Food Choices Due to Growing Awareness of Health

Participants, who were experienced in the workplace, described a move towards a healthier lifestyle and healthier food choices in more recent years. They reported that in the past, communal “cook-ups” had involved cooking with large amounts of added fat and salt and few vegetables. Today, despite large portion sizes, all groups believed their “cook-ups” were more balanced meals. Moreover, the workers today said they were more likely to bring in home-cooked meals or ingredients to cook with at work, while previously an energy-dense takeaway meal was eaten at the start of shifts. Factors contributing to this culture change included: improved cooking facilities at the stations, generational change with greater interest in health, fitness and cooking, regular work-health checks, improved health education, growing public awareness of men’s health issues, and improved awareness of potential health concerns in the ageing workforce.

The majority of participants, who were recruit firefighters, reported that they were more likely to prepare a balanced meal during shifts compared to their previous jobs, attributing this to the kitchen facilities, time available, and the social influence of others eating well.

“[My diet has] improved a little bit from my last job, just because there is a kitchen and you can cook and prepare your own food.”

—Focus group 2

Similarly, the presence of passionate health and fitness advocators within a station encouraged others to choose healthy foods at work, which occasionally carried over to home-life. As one participant explained:

“We actually have a very passionate personal trainer at our station… to the point where when he’s on shift people tend to eat healthier just because they don’t want to have to have that confrontation where they have to explain what they’re eating to him.”

—Focus group 5

## 4. Discussion

This mixed methods study found that shift schedule influenced food choices. It also highlighted that there are opportunities to modify the workplace environment to support healthier food choices of those working both at night and during the day and influence positive health behaviours by using a group/work culture approach. These findings are important for developing strategies in the workplace that will encourage behaviour change to promote healthy eating for those who work shifts. This is important given the data that suggest negative metabolic consequences of working night shift [3,5].

Food choices are key modifiable determinants of chronic diseases such as cardiovascular disease and type 2 diabetes. This study showed that amongst shift workers, the meal consumed during a day shift differed from the meal consumed on night shift, but there were no differences in overall energy intake (kJ/day), which is consistent with findings of a recent systematic review and meta-analysis [16]. The foods chosen at lunch were bread-based products, whereas the meals consumed at night were hot “home-cooked” style meals with a higher energy density (ED_energy_ kJ/g). Following this type of meal structure appeared important to the participants, and the opportunity to prepare a cooked meal and share with colleagues was associated with emotions of pride (Focus group 5). Even when shifts occurred at the weekend, this brought about different food choices than during the week, consistent with the usual variation displayed by those who work a standard working week pattern [25].

Snacking behaviour was different when on night shift compared with day shift. Both the focus groups and dietary data suggest that snacks were more likely to comprise discretionary foods at night. These foods are generally higher in energy, sugar, and fat and may be adverse for metabolic control at night. Habit and lack of healthy alternatives in the workplace were discussed in the focus groups as reasons for discretionary snack choices at night. A previous study of U.S. firefighters also described the availability of unhealthy snacks at their fire stations as a barrier to healthy eating at work, however, it was not indicated whether this was influenced by shift schedule [26]. Other studies have found that increased snacking is related to a change in food preferences at night and the use of sugar and/or caffeine to improve alertness [14,15,27]. A recent narrative review summarising studies that have examined dietary behaviours of shift workers supports the findings of our study that eating frequency, quality of diet, and energy distribution are impacted by shift work [28]. Workplaces should make a concerted effort to ensure that the snacks and food items supplied at work (both night and day) are nutritious.

It has been established that the health behaviours of a group influence the behaviour of an individual [29] and workplace environment is recognised as a strong predictor of individual dietary behaviours [30]. Participants in this study demonstrated a collegial approach to food selection, preparation, and consumption. This may be more evident in this working group due to the type of work activities they engage in, which require exceptional teamwork to prevent potentially devastating consequences. These participants have a group culture not only towards the duties they perform at work, but also to eating and, at times, sleeping as a group. Previous studies of employees working within a team have found that co-workers have an impact on individual health choices. Nurses have reported craving discretionary items after seeing their colleagues eating them, a finding mirrored in the present study [31]. Another study found that the attitude towards fitness among highly ranked firefighters had a strong influence on their crew’s physical activity and fitness [32]. The PHLAME (Promoting Healthy Lifestyles: Alternative Models’ Effects) Firefighters study demonstrated that group support for nutritious eating can result in group commitment towards health promoting behaviour [33]. This evidence supports the finding of the present study that co-worker attitudes and dietary choices can have both a positive and negative effect on health. The theoretical domains framework (TDF) is a single framework used to inform intervention design and fits within the Behaviour Change Wheel which enables characterisation of the target behaviour in terms of Capability, Opportunity, and Motivation (COM-B). To support translation of our outcomes into strategies promoting dietary change in shift workers, we have mapped these key findings to behaviour system COM-B [34] and theoretical domains framework change theory [35] (Table 5).

In a recent report that explored eating patterns and related lifestyle behaviours in shift workers in Ireland, personal (intrinsic) factors that influenced (dietary) behaviour included motivation to change, nutritional knowledge, and organisational and planning skills [36]. A study in paramedics (*n* = 15) in Australia identified a number of physiological factors that impacted food choice including fatigue, hunger, and physical health [17]. Similar to the current study, data from these two studies were collected through focus groups; however, in our focus groups, personal factors that impacted food choice did not emerge as a Theme in the data.

Strengths of the study include the mixed methods approach, which allows stronger conclusions to be drawn from this study through the affirmation of qualitative and quantitative findings [37]. Furthermore, the repeated use of a multiple-pass method to collect dietary data allowed for the more accurate reporting of dietary intake, although the small sample for the dietary assessment component could be considered a limitation. As participants opted into the study, a risk of sampling bias was introduced. Lastly, firefighters are a unique group of shift workers and, consequently, some of the findings of this study may not be generalizable to the broader Australian shift working population, but may have applicability to related occupations such as other emergency services, law enforcement, and hospital emergency departments that share similar unpredictable work demands and work closely in teams.

## 5. Conclusions

This study identified a number of factors that may contribute to an increase in unhealthy dietary behaviours in a shift-working population, including an increase in discretionary foods and lack of availability of healthy food choices at night. As shift workers are at an increased risk of developing obesity [1,2,38,39] and cardiovascular disease [3,4] compared to the non-shift-working population, these findings provide important data to inform workplace health policy to support improving the health of these at risk and vulnerable employees.

## Figures and Tables

**Table 1:** Questions used to guide semi-structured focus group discussions.

Question	Information Sought
Can you run me through what you would eat and drink at work on a day shift?	Usual dietary intake during a day shift
Where does your food come from on a day shift? For example, do you bring it from home, buy it at work…?	Source of food during a day shift
What influences these food choices on day shift? Do you ever find that you eat on a day shift for reasons other than hunger? If yes, what are these reasons? Why do eat at the times that you do?	Factors influencing food choices during a day shift
Can you now run me through what you would eat and drink at work on a night shift?	Usual dietary intake during a night shift
On a night shift, where does your food come from?	Source of food during a night shift
What influences the types of foods that you eat on night shift? Thinking about a night shift, what are the reasons for you choosing to eat when you do? Say you have just come back from a call at 2 a.m., will you have something to eat or go straight to bed?	Factors influencing food choices during a night shift
Recruit firefighters (<1 year): Since starting shift work, have there been any notable changes in what influences your food choices? *Experienced firefighters*: Have you noticed any dietary changes over your years of working with the Metropolitan Fire Brigade?	Long-term effects of shift work on dietary intake

**Table 2:** Characteristics of study participants.

	Focus Group *n* = 41	Dietary Recall ^c^ *n* = 19
Age (years)	36 (30, 52) ^a^	36 (29, 51)
Body mass index (BMI) (kg/m^2^)	26 (24.7, 27.8) ^a^	24.7 (23.4, 26.5)
Self-reported weight gain since starting shift work (kg)	24 (58.5) ^a^	9 (47.4)
Male gender (%) ^b^	40 (97.6)	18 (94.7)
Proportion aged: (%) ^b^		
<25 years	2 (4.9)	1 (5.3)
25–34 years	16 (39)	8 (42.1)
35–44 years	6 (14.6)	3 (15.8)
>45 years	17 (41.5)	7 (36.8)

^a^ Median (25th, 75th percentiles); ^b^ % of total group; ^c^ *n* = 18 also completed the focus groups.

**Table 3:** Themes identified from focus groups of rotating shift workers.

Themes	Descriptors
1. Shift schedule influences types of meals and snacks consumed at work.	Meals prepared at work: “Communal cook-ups” Meals bought during shift: Takeaway choices Meals brought to work from home Meals provided by the organisation Snacks during work hours
2. Dietary intake is affected (both positively and negatively) by the dietary choices and attitudes of co-workers.	Impact of others’ dietary choices Impact of co-workers’ attitudes toward food and health
3. Food choices during a shift are dependent on time availability and ease of access.	Non-hungry eating Impact of workplace protocol, structure and location Demands of the day’s tasks
4. Firefighters endeavour to make healthy food choices due to growing awareness of health within the brigade.	Preparing balanced meals together Cooking facilities Greater interest in health

**Table 4:** Median dietary intake of shift workers (*n* = 19) using 24 h dietary recalls.

	Total Daily Intake 24 h Period Includes Day Shift		Total Daily Intake 24 h Period Includes Night Shift		*p* Value *	Dietary Intake at Work (Day Shift 10 h)		Dietary Intake at Work (Night Shift 14 h)		*p* Value *
Energy (kJ/day)	11,491	(9986, 13,452)	10,350	(8519, 12,939)	0.295	6403	(4609, 7808)	5693	(4072, 6900)	0.171
Protein %EI	23.2	(19.5, 28)	21.4	(19.8, 24.2)	0.053	21.1	(19.4, 27.2)	23.1	(21.7, 25.2)	0.968
Total fat %EI	32.4	(27.6, 38.4)	33.0	(29, 36.8)	0.904	31.5	(28.8, 40.6)	29.2	(22.1, 36.7)	0.295
Saturated fat %EI	12.3	(9.7, 14.1)	12.5	(10.5, 13.5)	0.936	11.9	(10.4, 12.4)	11.9	(7.5, 14.2)	0.841
Carbohydrate %EI	38.9	(34.2, 44)	43.8	(36.5, 45.7)	0.117	43.6	(30.4, 46.4)	43.2	(36.5, 49.5)	0.277
Sugar %EI	15.5	(11.3, 19.7)	16.8	(14.2, 19.6)	0.036	17.8	(12.1, 19.2)	15.0	(10.9, 18.4)	0.494
Number of foods consumed	27.5	(21.5, 30)	25.0	(20, 30)	0.029	16.0	(12, 18)	11.5	(8, 15)	0.001
ED_all_ (kJ/g/day)	6.62	(6.16, 7.12)	7.36	(6.06, 8.14)	0.077	6.85	(5.69, 7.97)	6.61	(5.64, 7.55)	0.546
ED_solid_ (kJ/g/day)	6.56	(6.11, 6.8)	6.40	(6.04, 7.99)	0.117	8.68	(6.77, 9.55)	8.95	(7.14, 10.02)	0.421
ED_energy_ (kJ/g/day)	5.52	(4.72, 5.83)	5.73	(5.08, 6.88)	0.044	7.81	(6.45, 9.34)	7.37	(6.65, 9.55)	0.904

* Calculated using Wilcoxon signed-rank test. Significance level *p* < 0.05. All values are median (25th, 75th percentiles) %EI—percentage of total energy intake; ED_all_—all food and beverages; ED_solid_—solid foods only; ED_energy_—all solid foods, soups, milk as food, soups, milk as a drink and beverages containing > 21 kJ/100 g.

**Table 5:** Theoretical behaviour change components (COM-B) to address when implementing dietary changes for rotating shift workers.

COM-B Components		TDF Domain	Examples of Outcomes
Capability	Psychological	Knowledge Skills	Training/education in meal preparation and how to prepare for unexpected shift durations or tiredness
Opportunity	Physical Social	Environmental context and Resources Social influences	Provide meal preparation environments Ensure employer supplied meals and snacks are healthy Group commitment to healthy food environment
Motivation	Reflective	Social/Professional role and Identity Beliefs about consequences Optimism	Co-workers choices and attitudes affect dietary choices Knowledge of food choices and health outcomes—and that these health outcomes may differ with shift work Proud of having a well prepared dinner

COM-B: Capability, Opportunity, and Motivation (the COM-B system in the Behaviour change wheel), TDF: Theoretical Domain Framework.
